# Neuronally enriched microvesicle RNAs are differentially expressed in the serums of Parkinson’s patients

**DOI:** 10.3389/fnins.2023.1145923

**Published:** 2023-07-06

**Authors:** Morris A. Aguilar, Shauna Ebanks, Havell Markus, Mechelle M. Lewis, Vishal Midya, Kent Vrana, Xuemei Huang, Molly A. Hall, Yuka Imamura Kawasawa

**Affiliations:** ^1^Department of Veterinary and Biomedical Sciences, The Pennsylvania State University, University Park, PA, United States; ^2^Huck Institutes of the Life Sciences, The Pennsylvania State University, University Park, PA, United States; ^3^Department of Neurology, College of Medicine, The Pennsylvania State University, Hershey, PA, United States; ^4^Department of Pharmacology, College of Medicine, The Pennsylvania State University, Hershey, PA, United States; ^5^Department of Environmental Medicine and Public Health, Icahn School of Medicine at Mount Sinai, New York, NY, United States; ^6^Department of Biochemistry and Molecular Biology, The Pennsylvania State University, University Park, PA, United States; ^7^Institute for Personalized Medicine, College of Medicine, The Pennsylvania State University, Hershey, PA, United States

**Keywords:** Parkinson’s disease, exosome, microvesicle, small RNA, biomarkers, regression, random forest

## Abstract

**Background:**

Circulating small RNAs (smRNAs) originate from diverse tissues and organs. Previous studies investigating smRNAs as potential biomarkers for Parkinson’s disease (PD) have yielded inconsistent results. We investigated whether smRNA profiles from neuronally-enriched serum exosomes and microvesicles are altered in PD patients and discriminate PD subjects from controls.

**Methods:**

Demographic, clinical, and serum samples were obtained from 60 PD subjects and 40 age- and sex-matched controls. Exosomes and microvesicles were extracted and isolated using a validated neuronal membrane marker (CD171). Sequencing and bioinformatics analyses were used to identify differentially expressed smRNAs in PD and control samples. SmRNAs also were tested for association with clinical metrics. Logistic regression and random forest classification models evaluated the discriminative value of the smRNAs.

**Results:**

In serum CD171 enriched exosomes and microvesicles, a panel of 29 smRNAs was expressed differentially between PD and controls (false discovery rate (FDR) < 0.05). Among the smRNAs, 23 were upregulated and 6 were downregulated in PD patients. Pathway analysis revealed links to cellular proliferation regulation and signaling. Least absolute shrinkage and selection operator adjusted for the multicollinearity of these smRNAs and association tests to clinical parameters via linear regression did not yield significant results. Univariate logistic regression models showed that four smRNAs achieved an AUC ≥ 0.74 to discriminate PD subjects from controls. The random forest model had an AUC of 0.942 for the 29 smRNA panel.

**Conclusion:**

CD171-enriched exosomes and microvesicles contain the differential expression of smRNAs between PD and controls. Future studies are warranted to follow up on the findings and understand the scientific and clinical relevance.

## 1. Introduction

Parkinson’s disease (PD) is a neurodegenerative disorder characterized clinically by motor dysfunction (Fearnley and Lees, 1991) and pathologically by dopaminergic cell loss and Lewy inclusions in the substantia nigra (Fearnley and Lees, 1991). Despite effective symptomatic treatment, patients continue to experience progressive disability. There is a need to discover stable biomarkers to help diagnose disease and capture pathophysiological changes in live patients.

Exosomes and microvesicles are nanovesicles released into the extracellular environment by most cell types for intercellular communication (Raposo and Stoorvogel, 2013), and their molecular cargo is protected from enzymatic breakdown (Banack et al., 2020). The size of exosomes ranges from 40–200 nm, and the exosomes contain proteins and nucleic acids secreted from host cells to the surrounding or distant body parts (Trams et al., 1981; Yuan and Li, 2019; Banack et al., 2020). Exosome isolation methods often include microvesicles that are between 200 nm and 1,000 nm (Raposo and Stoorvogel, 2013; Cao et al., 2017; Hill, 2019). Those microvesicles can be generated by −80°C storage that induces exosome fusion that yields vesicles >200 nm (Gelibter et al., 2022). Nucleic acids found in exosomes and microvesicles include long noncoding RNA, messenger RNA, micro RNA, ribosomal RNA, ribosomal pseudogenes, and genomic DNA (Srivastava et al., 2015; Liu et al., 2020; Sproviero et al., 2021). They can cross the blood–brain barrier (Alvarez-Erviti et al., 2011) into peripheral blood, thus carrying molecular markers originating from the central nervous system (Kalluri and LeBleu, 2020; Serpente et al., 2020). Small RNAs (smRNAs), including micro RNAs (miRNAs) and piwi-interacting RNAs (piRNAs), have been isolated from exosomes and microvesicles. SmRNAs (21–26 nucleotides) can regulate post-transcriptional gene expression via silencing through homologous sequence interactions (Finnegan and Matzke, 2003). Thus, exosomes and microvesicles contain smRNAs and serve as reservoirs rich with disease-related information to inform pathophysiology and potential biomarker development.

Exosomes and microvesicles enable the transmission of biomolecules between cells, thus, they may play a role in spreading or modulating disease processes (Asai et al., 2015; D’Anca et al., 2019; Jiang et al., 2019). Consistent with this idea, misfolded proteins associated with neurodegenerative diseases, such as α-synuclein (α-syn), tau, and amyloid β (Aβ), can be transported through exosomes and microvesicles (Asai et al., 2015; Jiang et al., 2019). Additionally, miR-125, miR-210, miR-450b, and miR-669b from exosomes and microvesicles promote signaling pathways triggering manganese-dependent α-syn overexpression and deposition, the protein characteristic of PD pathogenesis (Danzer et al., 2012; Harischandra et al., 2018). Interestingly, exosomal miRNA-7 also reduces the expression of α-syn (Junn et al., 2009). Exosomes and microvesicles can enrich and stabilize miRNAs by preventing degradation by nucleases widely present in body fluids (Chen et al., 2012; Wang and Zhang, 2020). Compared with the direct detection of biomarkers such as DJ-1, oxDJ-1, α-syn, and miRNA in the CSF or blood, exosome and microvesicle detection of these PD-related biomarkers has been reported to be more stable and reliable, and a better reflection of the PD disease state (Hartfield et al., 2012; Saito, 2017). Differentially expressed exosomal miRNAs effectively predicted the PD phenotype via univariate linear regression models (Cao et al., 2017). In another study, expression levels of serum exosomal miRNAs were increased significantly in PD patients and multivariate linear regression models predicted PD status better than univariate regression models (Barbagallo et al., 2020). Together, these studies suggest that these nucleic acids may play a major role in PD pathogenesis and potentially be biological markers of the disease.

Although past studies have attempted to identify circulating smRNAs as biomarkers of PD (Li and Le, 2020; van den Berg et al., 2020; Nies et al., 2021), the findings have been inconsistent (Li and Le, 2020). This might result from differences in smRNA expression profiling according to sample types such as whole blood (Vaz et al., 2010), cell-free serum, or plasma (Tsujiura et al., 2010; Sheinerman et al., 2012). It is challenging to differentiate disease-specific smRNAs since they are derived from any organ in contact with blood (Cheng et al., 2014). CD171 immunocapture of neuronal exosomes and microvesicles in blood plasma previously detected differences in α-syn [a hallmark of PD (Spillantini et al., 1997)] levels between PD and controls (Niu et al., 2020). The specific capture of CD171 exosomes and microvesicles from blood serum has been demonstrated for neurocognitive diseases such as Alzheimer’s (Fiandaca et al., 2015; Pace et al., 2019; Serpente et al., 2020), opiate addiction (Fiandaca et al., 2015; Kumar et al., 2021), and amyotrophic lateral sclerosis (Banack et al., 2020). Recent literature demonstrated that increased neuronal exosomes and microvesicles from saliva had been detected in PD patients via CD171 immunoprecipitation capture (Rani et al., 2019). No study, however, has examined smRNA cargo in neuronal exosomes and microvesicles in blood serum from PD patients. In this study, we applied a previously published method to amplify smRNAs from neuronal-origin exosomes and microvesicles in serum using a highly expressed neuronal marker protein, CD171 (Shi et al., 2014; Banack et al., 2020; Jiang C. et al., 2020) (also known as L1CAM). We hypothesized that a panel of captured exosomal smRNAs would differ significantly between PD and control participants and discriminate PD subjects from controls.

## 2. Materials and methods

### 2.1. Subjects

We obtained demographic and clinical data from 40 control and 60 PD subjects matched for age and sex who participated in the National Institute of Neurological Disorders and Stroke PD Biomarkers Program (NINDS PDBP). According to published clinical criteria, movement disorder specialists confirmed PD diagnosis (Jamalabadi et al., 2016). All subjects were free of major/unstable medical issues or neurological conditions other than PD. All subjects gave written informed consent. The study was conducted following the Declaration of Helsinki and reviewed and approved by the Penn State Hershey Institutional Review Board.

Demographic data were collected, including age, sex, education, smoking history, antiparkinsonian drug treatment, and clinical parameters. Education and smoking data were included because of their suggested protective roles in PD (Gorell et al., 1999; Kotagal et al., 2015). PD duration was obtained from the subject’s history, with onset defined as the first diagnosis by a medical professional. Motor symptoms were assessed using the Movement Disorders Society Unified PD Rating Scale motor subscale (MDS-UPDRS-III) and disease stage by Hoehn and Yahr (1967). Depression was evaluated using the Hamilton Depression Rating Scale (HDRS) (Hamilton, 1960) and cognition by the Montreal Cognitive Assessment (MoCA) (Nasreddine et al., 2005). The University of Pennsylvania Smell Identification Test (UPSIT) (Doty et al., 1984) assessed olfactory function. These clinical tests describing PD also reaffirm the diagnosis of the PD participants. Three pairs of discordant monozygotic twins (one has PD but his/her monozygotic sibling does not) were included in the study.

### 2.2. Blood sample collection and serum preparation

Blood samples were collected from subjects after an 8–12 h overnight fast. Within 30–60 min of the blood draw, samples were centrifuged at 1,500 x g for 15 min at 4°C. One mL aliquots of the supernatant were pipetted into cryovials on ice and then stored at −80°C. Samples were thawed and 100 μL was pipetted into a separate cryovial tube for the following assays.

For the study, we used blood serum over plasma to reduce the presence of proteins, lipids, and sugars in the final solution. Previous research has shown that clotting factors in plasma can contribute to the variability of exosome and microvesicle concentration (Muller et al., 2014).

### 2.3. Collection of neuronal origin exosomes and microvesicles

**Figure 1:**
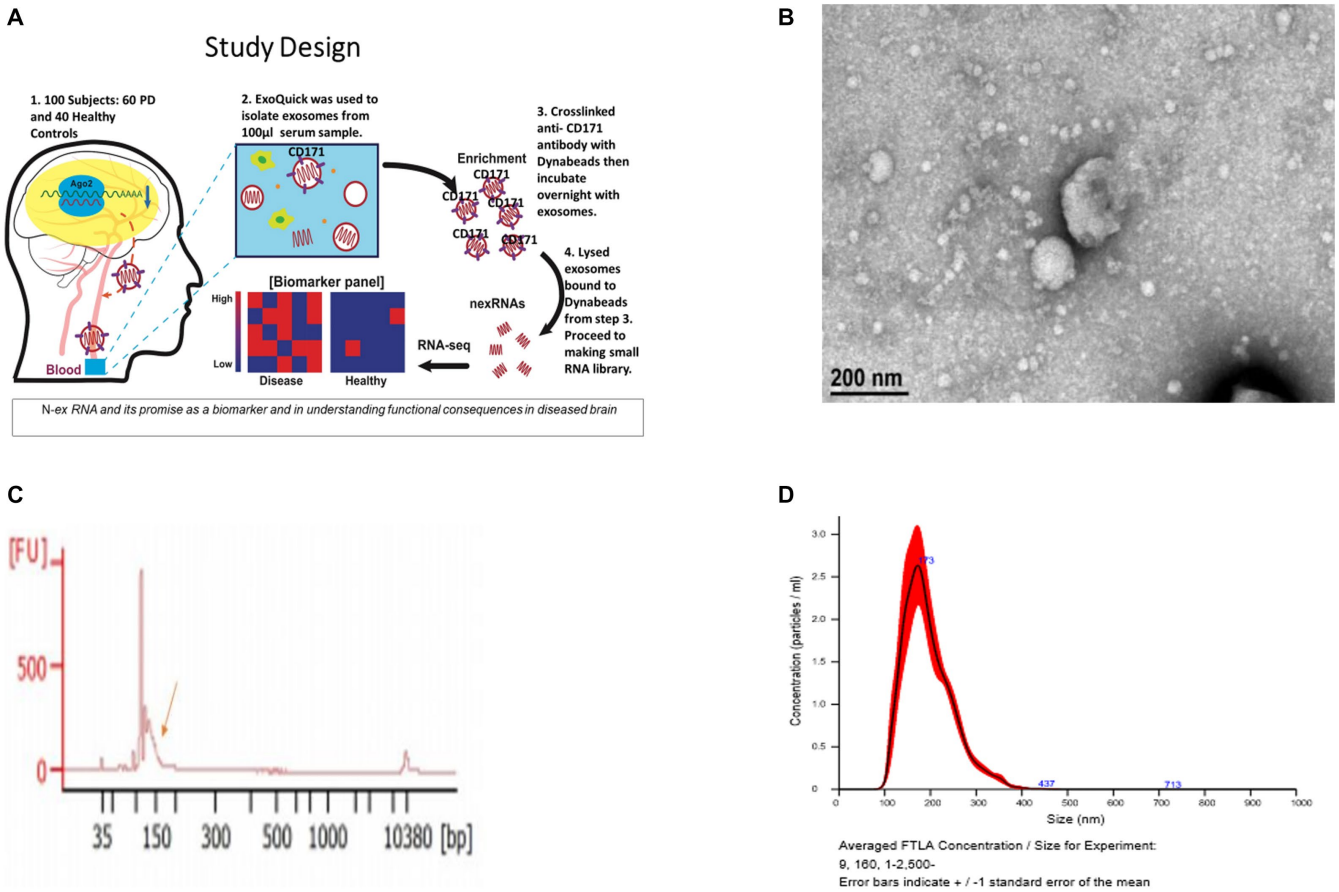
Validation of exosomes isolated from serum sample via bioanalyzer, transmission electron microscopy (TEM), and nanoparticle Tracking Analysis (NTA). **(A)** Schematic of the study design. **(B)** Morphological characterization of exosomes isolated from serum samples by transmission electron microscopy. Bar, 200 nm. **(C)** Bioanalyzer report of smRNA fragment length distribution. **(D)** Graph generated by NanoSight showing concentration and average size of the vesicles after ExoQuick precipitation showing exosomes collected in the 100–400 nm range.

The exosomes and microvesicles were purified from the blood samples to separate free circulating smRNAs from smRNAs contained within the vesicles of interest (Otake et al., 2019). ExoQuick™ Exosome Precipitation Solution kit (Systems Biosciences) was used to extract exosomes and microvesicles from the samples (Figure 1A). Serum (100 μL) was centrifuged at 3,000 × g for 15 min at room temperature to remove cells and cellular debris. The supernatant was transferred to a sterile vessel and 25.2 μL of ExoQuick™ Exosome Precipitation Solution was added, refrigerated at 4°C for 30 min, and centrifuged at 1,500 g for 30 min at 4°C. The supernatant was aspirated, leaving the exosomes and microvesicles as a white pellet. Another centrifugation at 1,500 × g for 5 min was done to remove traces of ExoQuick™ by aspiration. The exosome pellet was resuspended in 100 μL phosphate-buffered saline.

The resuspended pellets were analyzed to confirm that the purified exosomes and microvesicles had the expected size and concentration measurements. To measure concentration and size, exosomes and microvesicles from 3 control and 3 PD subjects were characterized using Transmission Electron Microscopy (TEM) and NanoSight Tracking Analysis. The TEM measurements were used to confirm that the vesicles in the buffer had an expected size of 40–200 nm (Tomlinson et al., 2015) and microvesicles ranging from 200–1,000 nm. Only three from each group were randomly selected due to time and cost. Our result aligns with the expected range (100–400 nm) of the vesicle size distribution via ExoQuick™ (Caradec et al., 2014).

Exosomes and microvesicles with neuronal origins were extracted from the solution by capturing exosomes and microvesicles expressing the neuron-specific marker, CD171 [L1 cell adhesion molecule (Fiandaca et al., 2015)]. A biotinylated anti-human CD171 antibody (eBio5G3, Affymetrix) was used to bind vesicles expressing CD171. Then streptavidin-conjugated magnetic beads (#10608D, Thermo Fisher Scientific) were added to the solution to bind to the CD171 antibody. Neuronal exosomes and microvesicles bound to anti-CD171 and streptavidin beads were pulled down magnetically. The captured neuronally derived exosomes and microvesicles were lysed with IGEPAL^®^ CA-630 (Sigma-Aldrich) to free the smRNAs into solution; IGEPAL^®^ was added to 1% of the final concentration. These exosomal neuronally derived smRNAs were used for sequencing library preparation.

### 2.4. SmRNA-sequencing (RNA-Seq)

SmRNA sequencing libraries were generated using the CleanTag^®^ Small RNA Library Prep Kit (TriLink Biotechnologies) for downstream smRNA expression analysis (Tomlinson et al., 2015; Olivares et al., 2020). Individually barcoded libraries were mixed equimolarly and subjected to sequencing with technical duplicates on an Illumina NovaSeq 6000. We used Pearson’s product–moment correlation to evaluate technical replicates to indicate that the read counts between each sequencing run on the NovaSeq 6000 run were consistent (Otake et al., 2019; Supplementary Figure S1).

### 2.5. Quantity filtering and read alignment of smRNA sequencing reads

Before expression analysis, we quality-filtered and aligned the smRNA sequencing reads. FASTX-Toolkit (Gordon and Hannon, 2010) was used for quality filtering and adapter clipping from the raw sequences. Oasis 2.0 suite was then used to align and count raw reads of all expressed smRNAs (Rahman et al., 2018). Subjects whose total read count fell into the lowest quartile of all read counts were removed. The first quartile (5,045 reads) was used as a cut-off threshold. This process removed 27 samples, 15 PD and 12 controls. The final dataset consisted of 28 controls and 45 PD cases. The PD samples were filtered to include smRNAs with at least five non-zero reads. We used the Degust web-based RNA sequencing data visualization tool to create an expression matrix and multidimensional scaling (MDS) plot.^1^ The resulting expression data matrix contained 108 smRNAs and *n* = 73 subjects visualized into an MDS plot (Powell et al., 2019).

### 2.6. Differential smRNA expression analysis and smRNA clinical correlations

Differential expression analysis was performed to detect how PD patients’ neuronal exosomal smRNAs may be perturbed by the disease. We also explored how anti-PD drugs can affect smRNA expression in PD participants. The analysis used quasi-likelihood functionality in edgeR (Chen et al., 2014), available in the Degust tool (Powell et al., 2019) to identify differences between PD and control cases. These differentially expressed smRNAs were clustered according to log_2_ of counts per read (CPM) and plotted in a heatmap using the *ComplexHeatmap* R package (Gu, 2021). *p*-values were controlled for multiple comparisons using false discovery rate (FDR); FDR < 0.05 was considered significant.

We tested the association between smRNA expression and clinical parameters (i.e., age at visit, disease duration, smoking, Hoehn and Yahr Stage, HDRS, total MDS-UPDRS-III, MoCA, and education) using least absolute shrinkage and selection operator (LASSO) followed by linear regression. The multicollinearity of smRNAs (Barbagallo et al., 2020; Quintanilha et al., 2021) was adjusted using feature selection via LASSO [Scikit-learn (Pedregosa et al., 2011), Python package] for each clinical parameter to reduce false positives and the number of statistical tests. For each clinical parameter, the optimal LASSO penalty value (lambda) was determined with 5-fold cross-validation with a 60 and 40% random split between training and testing; this aided in avoiding a lambda value from an overfit linear model. The set of smRNA predictor variables that did not have their coefficients reduced to 0 by LASSO was selected for each clinical outcome. This set of smRNAs for each outcome was tested for association with linear regression and multiple test corrected with Bonferroni adjustment.

### 2.7. Detection of expected PD marker, miR-26b-5p

Quantitative real-time polymerase chain reaction (qRT-PCR) was performed on one of the differentially expressed smRNAs to confirm that the RNA-Seq results contained an expected PD marker. The miR-26b-5p is an important marker strongly discriminating between PD and control groups and has been patented for PD diagnosis (Keller et al., 2015; Mushtaq et al., 2016; Fyfe, 2020). This assay used five PD and five control subjects selected randomly. The smRNA Illumina libraries were used as sample input, and qRT-PCR conditions were: 95°C for 10 min, 40 cycles of 15 s of denaturation at 95°C, and 30 s of annealing/elongation at 55°C using a QuantStudio 12 K Flex Real-Time PCR System (Thermo Fisher Scientific). The forward primer sequence was TCAAGTAATTCAGGATAGGT, and the reverse primer sequence was GAGTTCCTTGGCACCCGA. Mean fold gene expression was calculated with the 2^-ΔCT^ method (Livak and Schmittgen, 2001).

### 2.8. Logistic regression and random forest classification of PD using a smRNA panel as predictors

Logistic regression (LR) and random forest (RF) classification models were created to identify smRNAs predictive of the PD phenotype. The LR and RF classification methods used the smRNA read counts as filtered, aligned, and normalized predictors. For the LR and RF models, 60% of the samples were selected randomly for training and 40% for testing. Univariate LR models were created with the *glm2* package (EBSCOhost, 2022) for R to determine which smRNA reads predicted PD. Given this analysis’s small sample size, including non-prognostic covariates can decrease power and inflate false-positive rates (Raab et al., 2000; Bursac et al., 2008; Kahan et al., 2014). We considered demographic variables (age, sex, education, and smoking) as covariates if they met the following criteria: (Raab et al., 2000; Bursac et al., 2008; Kahan et al., 2014) (1) unbalanced values between groups and (2) not significantly related to the PD outcome with univariate association tests. The *pROC* package (Robin et al., 2011) for R was used to determine the area under the receiver operating characteristic curves (AUC) for this smRNA panel in the LR and RF models; RF-based classification was performed with 5-fold cross-validation using the *randomForest* R package (Liaw and Wiener, 2002). The RF model used 29 smRNA predictors that were significantly differentially expressed. The importance of the predictors relative to each other in the RF model was determined by calculating the Mean Decrease in Gini to reflect how well a variable discriminated PD and control subjects: a larger *MeanDecreaseGini* value suggests the variable plays a greater role in the classification process (Louppe et al., 2013).

### 2.9. Database search for smRNA function and regulation pathways

Pathway analysis describes how these smRNAs function in the context of genes and proteins. The statistically significant differentially expressed smRNAs were used as input for pathway analysis. The functional regulatory networks were evaluated using an Ingenuity Pathway Analysis (Krämer et al., 2014) (IPA) (QIAGEN Inc.) with a miRNA Target Filter.

## 3. Results

### 3.1. Demographic and clinical characteristics of study participants

Demographic and clinical characteristics were tested for statistically significant differences between the PD and control participants to identify covariates to include in the downstream differential expression analysis. There was no significant difference in age (*p* = 0.90), sex distribution (*p* = 0.70), education level (*p* = 0.16), or smoking history (*p* = 0.75) between the groups (Supplementary Table S1). Given that these variables were balanced, they did not meet the criteria to be included as covariates. PD subjects had higher MDS-UPDRS-III (*p* < 0.0001) and Hamilton depression scores (*p* < 0.0001) than controls and lower MoCA (*p* = 0.0003) and UPSIT scores (*p* < 0.0001), all of which survived Bonferroni correction (*p* < 0.0065) for the eight demographic and clinical characteristics tests. Hoehn and Yahr scale scores were higher in PD subjects than in control subjects (*p* = 0.0001). Scores ranged from 1–5 and had a mean of 2.2 for PD participants. Most PD subjects (*n* = 36) had a disease duration of <10 years (Table 1). Among the PD patient samples that passed smRNA sequencing read quality control (*n* = 46), 42 were on antiparkinsonian medications and four were drug-naive.

**Table 1:** Demographic and clinical characteristics of study participants.

	Control (*n* = 40)	PD (*n* = 60)	*P* value
Age (years; Mean ± SD)	66.3 ± 10.9	66.6 ± 9.9	0.90
Sex (Male/Female)	20/20	33/27	0.77
Education	17.2 ± 2.9	16.4 ± 3.1	0.16
Smoking (Yes/No/^*^)	10/30/0	18/40/2	0.75
MDS-UPDRS Score-III	5.3 ± 5.6	31.0 ± 23.6	<0.0001
Hamilton Depression Scale	2.3 ± 3.0	7.2 ± 5.2	<0.0001
MoCA	25.7 ± 2.42	23.0 ± 4.5	0.0003
UPSIT	31.7 ± 6.26	20.1 ± 8.13	<0.0001
Hoehn and Yahr Scale	0.38 ± 0.90	2.2 ± 1.16	<0.0001
Antiparkinsonian drugs (Yes/No)			
Whole cohort	NA	56/4	NA
After sequencing read quality control	NA	42/4	>0.7
PD Duration (Years)	<10 years	10–15 years	>15 years
Number of PD Patients	36	11	13

Data represent the mean ± standard deviation unless otherwise indicated. ^*^Indicates data were not available for subjects. PD, Parkinson’s disease; MDS-UPDRS-III, Movement Disorders Society Unified PD Rating Scale motor subscore; MoCA, Montreal Cognitive Assessment; UPSIT, University of Pennsylvania Smell Identification Test. NA, Not applicable.

### 3.2. Characterization of isolated serum exosomes and microvesicles by NanoSight

Vesicle size and concentration were used to confirm the presence of exosomes and microvesicles and to detect vesicle size differences between groups. The average sample concentration was 0.173 vesicles per μL of serum and the vesicle size was 100–400 ± 5 nm (Figures 1B–D). The average vesicle size was 173 nm, and the size ranges are aligned with expected exosome (40–200 nm) and microvesicle (200–1,000 nm) sizes. There was no significant difference (*p* > 0.05) in vesicle concentration or size between PD and controls. Insignificant differences in vesicle sizes indicate that the exosomes and microvesicles have similar morphology between PD and control groups.

### 3.3. SmRNA expression profile

**Figure 2:**
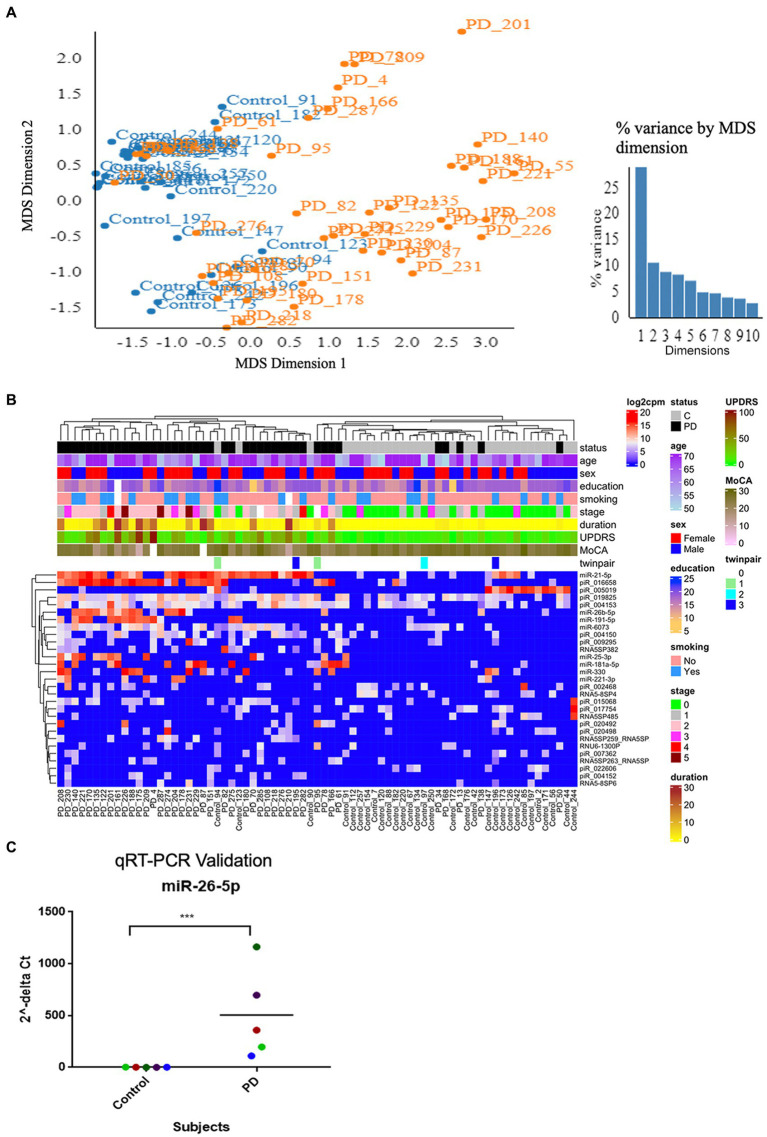
Altered smRNAs profiles in neuronally-enriched serum-derived exosomes of PD by RNA-Seq Analysis. **(A)** MDS analyzes the smRNA read counts from PD and control subjects. **(B)** Unsupervised hierarchical clustering of the 29 differentially expressed smRNAs (rows) for the 45 PD and 28 control samples (columns). The sample ID and the top column in the annotation bars indicating the disease state of each subject in a color code (grey for controls and black for PD cases) are shown at the top of the plot. The other annotation bars illustrate age, sex, education, smoking status, disease stages, disease duration, MDS-UPDRS, MoCA, twin pairs, and the expression level of smRNAs across all samples. Not available values are shown in white. **(C)** qRT-PCR was used to confirm the level of miR-26b-5p. Relative levels of one miRNA (miR-26b-5p) in the serum of PD cases and controls were determined by qRT-PCR. Each point represents the mean of triplicate samples. ***p < 0.001.

The quality filtered and aligned reads were analyzed to detect clustering separation between groups, testing confounding effects, and differential expression data. We used multidimensional scaling (MDS) as an exploratory analysis tool to identify patterns among the aligned reads. The MDS plot of filtered smRNA reads revealed differences in clustering separation between PD and control subjects (Figure 2A). There was no significant clustering in the hierarchical clustering analyses of smRNA reads regarding age, sex, education, or smoking history (Figure 2B). However, there was hierarchical clustering among the PD samples and the duration of PD, MDS-UPDRS-III, and MoCA (Figure 2B). After FDR multiple test correction, differential expression analysis between PD and control samples resulted in 29 smRNAs being significant for differential expression (22 up- and 7 down-regulated in PD subjects, FDR < 0.05) (Table 2). The unsupervised hierarchical clustering analysis of these 29 smRNAs grouped the majority of the PD samples to the left and control samples clustered predominately on the right of the heatmap (Figure 2B). Of the 29 smRNAs, there was no differential expression between those on or never on antiparkinsonian medications (*p* > 0.7) (Supplementary Table S1).

**Table 2:** Target genes and associated regulatory networks for differentially expressed miRNAs between PD and controls.

Upregulated in PD	Log_2_ (Fold-Change)	FDR	Average expression	*P-value*
hsa-miR-26b-5p	9.804	5.74E-7	12.048	8.16E-9
RNA5SP382	7.874	5.74E-7	9.955	1.59E-8
hsa_piR_009295	6.851	9.25E-7	9.451	3.42E-8
hsa_piR_020498	5.993	7.14E-5	9.634	4.63E-6
hsa_piR_020492	4.851	1.804	9.979	2.84E-4
hsa-miR-181a-5p	4.758	7.64E-3	13.196	1.42E-3
hsa_piR_016658	4.702	2.70E-3	14.593	4.51E-4
hsa-miR-25-3p	4.119	2.01E-2	11.630	4.37E-4
hsa-miR-191-5p	3.987	2.36E-2	12.683	5.68E-3
hsa_piR_004153	3.718	5.65E-4	9.442	7.85E-5
p-hsa-miR-330	3.550	4.36E-2	12.791	1.17E-2
hsa-miR-6,073	3.464	1.06E-4	8.159	9.83E-6
hsa-miR-221-3p	3.406	2.36E-2	9.901	5.51E-3
hsa-miR-21-5p	3.177	2.79E-2	14.211	6.99E-3
RNA5SP259_RNA5SP25	3.175	1.42E-6	6.693	6.56E-8
hsa_piR_004150	2.898	2.99E-4	7.369	3.53E-5
RNU6-1300P	2.823	2.99E-4	6.928	3.84E-5
hsa_piR_019825	2.731	3.01E-3	9.155	5.3E-4
hsa_piR_015068	2.400	1.70E-3	7.000	2.52E-4
RNA5-8SP6	2.248	2.99E-4	6.591	3.88E-5
hsa_piR_004152	1.981	2.4E-4	6.493	2.44E-5
RNA5SP263_RNA5SP26	1.893	2.01E-2	6.531	4.46E-3

Down-regulated in PD	Log_2_ (Fold-Change)	FDR	Average Expression	*P-value*
hsa_piR_005019	−7.375	5.03E-6	12.808	2.80E-7
hsa_piR_017754	−5.959	7.37E-5	8.617	5.46E-6
RNA5-8SP4	−3.011	5.74E-7	6.665	1.46E-8
hsa_piR_002468	−2.723	1.80E-2	9.047	3.67E-3
RNA5SP485	−2.656	8.48E-5	6.785	7.06E-6
hsa_piR_022606	−1.575	8.74E-3	6.443	1.7E-3
hsa_piR_007362	−1.169	4.37E-2	6.470	1.15E-2

### 3.4. Detection of a known PD marker, hsa-miR-26b-5p, via qRT-PCR

We used a known differentially expressed PD marker (Keller et al., 2015; Mushtaq et al., 2016; Fyfe, 2020), hsa-miR-26b-5p, as a positive control when detecting differentially expressed smRNAs. The qRT-PCR detected an hsa-miR-26b-5p expression significantly higher in PD than in control subjects (*p* < 0.001) (Figure 2C). This result indicated that the sequencing runs could capture smRNA signatures that differentiate PD and control patients.

### 3.5. Association of smRNAs with clinical parameters

LASSO was used to select the clinical parameters for association tests because of the expected multicollinearity of smRNAs. LASSO selected among the 29 differentially expressed smRNAs those to include in the regression association analysis for each clinical outcome (Table 3). Age at visit, disease duration, MoCA, total MDS-UPDRS, and education (years) outcomes were not tested for association because LASSO reduced the coefficients of the 29 smRNAs to zero for each outcome; this suggested these clinical parameters are affected by the multicollinearity of the 29 differentially expressed smRNAs. Hoehn and Yahr stage outcome included hsa-miR-21-5p as a predictor in the regression but did not yield statistically significant results (*p* = 0.227). Hamilton Depression Scale included 7 smRNAs [RNA5SP382 (*p =* 0.891), hsa-miR-181a-5p (*p =* 0. 840), hsa_piR_016658 (*p =* 0.129), hsa-miR-25-3p (*p =* 0.624), hsa-miR-191-5p (*p =* 0.139), hsa-miR-21-5p (*p =* 0.923), and hsa_piR_005019 (*p =* 0.156)] in the regression model. Hoehn and Yahr Stage and Hamilton Depression Scale were not associated significantly with the smRNAs having coefficients greater than zero [Bonferroni adjusted (α = 0.05) significance threshold (7.143 × 10^−3^) for 8 tests].

**Table 3:** Association of smRNAs with clinical parameters.

LASSO selected predictor variables	Coefficient value	*p*-value	Clinical parameter	Control range	PD range
-	-	-	Age at Visit	43–86	43.6–93
-	-	-	Disease Duration (years)	0	0–32
hsa-miR-21-5p	1.96E-04	0.227	Hoehn and Yahr Stage	0–3	1–5
-	-	-	MoCA	21–30	9–29
RNA5SP382	−3.01E-04	0.891	Hamilton Depression Scale	0–12	0–21
hsa-miR − 181a-5p	2.81E-04	0.840			
hsa_piR_016658	1.36E-03	0.129			
hsa-miR-25-3p	-1.25E-03	0.624			
hsa-miR-191-5p	−4.70E-03	0.139			
hsa-miR-21 − 5p	-5.10E-05	0.923			
hsa_piR_005019	−2.69E-03	0.156			
-	-	-	Total UPDRS	0–27	3–98
-	-	-	Education (Years)	8–23	7–21

LASSO was used to select the smRNAs for regression to test the association between smRNA expression levels and clinical parameters: age at visit, disease duration, Hoehn and Yahr stage, MoCA, Hamilton depression score, total MDS-UPDRS, and education (years). PD clinical parameters are listed along with the smRNAs tested for association (of the 29 smRNAs in the panel expressed differentially in PD and control subjects). The outcomes without predictor variables had all of the coefficients of the 29 smRNA variables shrunk down to zero. They are represented as a dash, thereby not having any smRNA predictors tested for association via regression.

### 3.6. Assessing predictive value with logistic regression and random forest analyses

**Figure 3:**
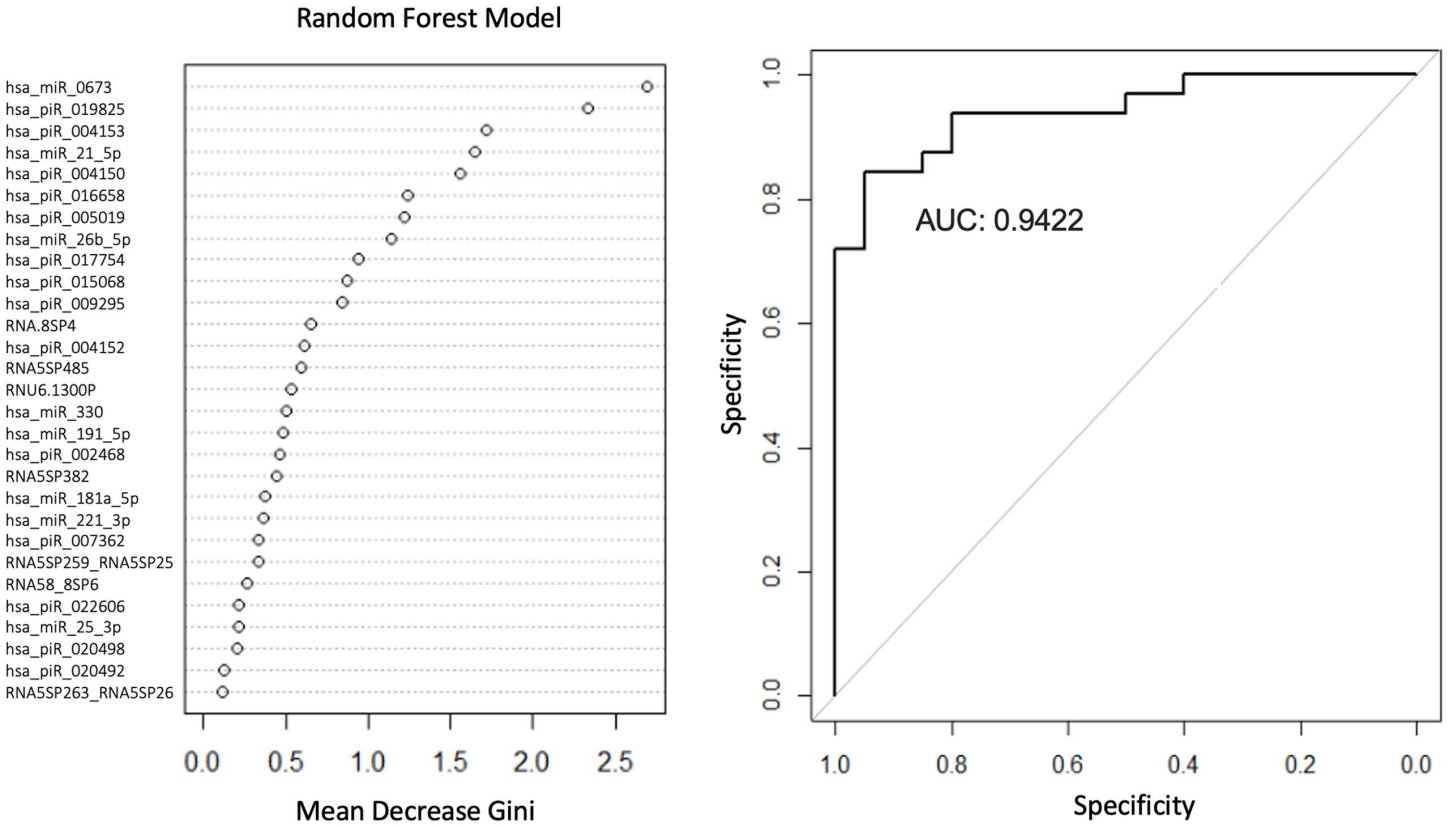
Random forest model diagnostic values and AUC curves. Variable importance plots obtained from Random Forest (RF) in R show the top smRNAs ranked based on the mean decrease in Gini coefficients. The ROC curve plot and the AUC value display the RF model’s performance in discriminating between PD and controls.

We used LR and RF analyses to identify which of the 29 statistically significant differentially expressed smRNA have predictive value for the PD phenotype. The AUC values represent performance on the test set. An AUC >0.5 suggests the model discriminates better between PD and control subjects than random chance. Univariate LR revealed an AUC of the ROC ≥0.74 for the following smRNAs: hsa-miR-6,073, hsa_piR_016658, hsa_piR_019825, and hsa-miR-21-5p (Table 4). Seven smRNAs (hsa_piR_020498, hsa_piR_007362, hsa_piR_017754, RNA5SP485, hsa_piR_022606, RNA5-8SP4, and hsa_piR_005019) each had an AUC < 0.5; these were not suitable for predicting PD and indicated small expression differences between control and PD subjects (Jamalabadi et al., 2016). The RF model trained on the 29 smRNAs generated an AUC of 0.942. The top 10 smRNAs in descending order of Gini coefficients were: miR_6,073, hsa_piR_019825, hsa_piR_004153, hsa.miR.21.5p, hsa_piR_004150, hsa_piR_016658, hsa_piR_005019, hsa.miR.26b.5p, hsa_piR_017754, and hsa_piR_015068 (Figure 3). The larger the *MeanDecraseGini* indicates that the variables are important in discriminating between the PD and control groups.

**Table 4:** Logistic regression AUC of 29 smRNAs.

smRNA marker	AUC	smRNA marker	AUC
hsa-miR-6,073	0.781	hsa_piR_004152	0.599
hsa_piR_016658	0.767	RNA5SP263_RNA5SP26	0.564
hsa_piR_019825	0.752	RNA5-8SP6	0.563
hsa-miR-21-5p	0.743	hsa-miR-221-3p	0.561
hsa_piR_004153	0.733	hsa_piR_020492	0.560
hsa_piR_004150	0.725	RNU6-1300P	0.546
hsa-miR-26b-5p	0.691	hsa_piR_002468	0.505
hsa_piR_015068	0.686	hsa_piR_020498	0.444
hsa_piR_009295	0.642	hsa_piR_007362	0.438
hsa-miR-191-5p	0.637	hsa_piR_017754	0.389
hsa-miR-25-3p	0.635	RNA5SP485	0.382
p-hsa-miR-330	0.631	hsa_piR_022606	0.378
RNA5SP382	0.622	RNA5-8SP4	0.359
RNA5SP259_RNA5SP25	0.616	hsa_piR_005019	0.343
hsa-miR-181a-5p	0.614		

Area under the curve (AUC) values were used to gauge the ability of the univariate LR models to discriminate between PD and control subjects using the 29 smRNA panel.

### 3.7. Targets and roles of miRNA

**Figure 4:**
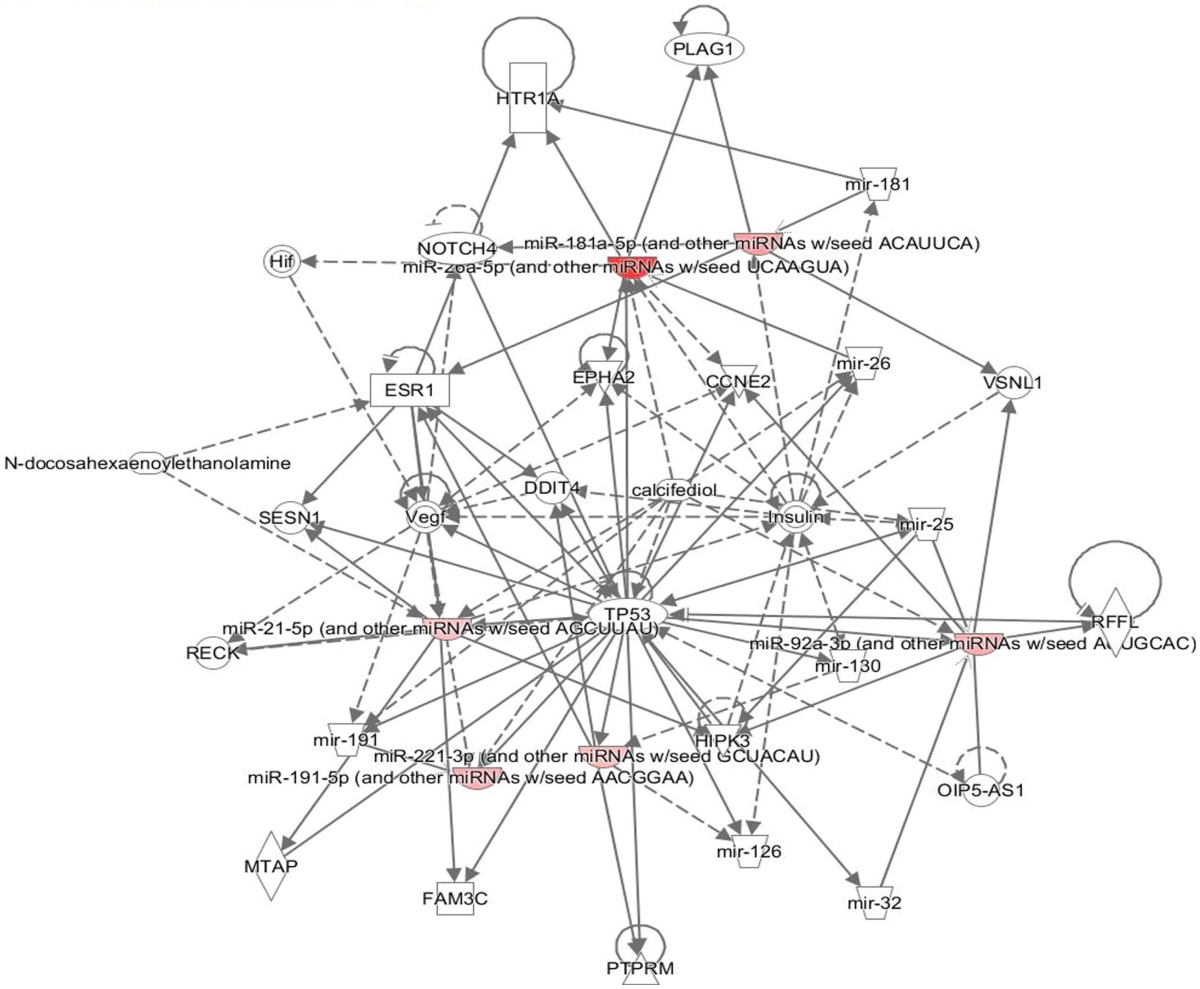
Gene pathways. The network displays the biological effects of the smRNAs on genes and other smRNAs. The edges with a solid arrowhead represent the direction of effects, and a blunted arrowhead represents inhibition. The input for the gene path analyses were the 29 differentially expressed smRNAs that were statistically significant.

The neuronally derived exosomal smRNAs were the only high throughput biomolecular data we measured, but pathway analysis aids in finding known associations between the smRNAs and other biological domains. The IPA functional network analysis used the 29 differentially expressed smRNA to build a network of genes and proteins that contextualize the function of the smRNAs. From the 29 differentially expressed smRNAs, the IPA network analysis displayed an association network (Figure 4) of smRNA targets that included *VNSL1* (calcium-mediated signaling), *TP53* (RNA-protein covalent cross-linking), *DDIT* (role in neuronal cell death), and *HTR1-A* (regulation of dopamine) (Table 5).

**Table 5:** Ingenuity pathway analysis (IPA) of targeted genes.

Gene	Full name	UNIPROT ID	Molecular function	Cellular component	Biological process
*VNSL1*	Visinin-like protein 1	P62760	Calcium ion binding	Cytosol	Calcium-mediated signaling
*Tp53*	Cellular tumor antigen p53	P04637	DNA strand annealing activity	Nuclear matrix	RNA-protein covalent cross-linking
*DDIT4*	DNA damage-inducible transcript 4 protein	Q9NX09	Required for normal neuron migration during embryonic brain development.	Cytosol	Plays a role in neuronal cell death
*HTR1-A*	5-hydroxytryptamine receptor 1A	P08908	G protein-coupled receptor for 5-hydroxytryptamine (serotonin)	Cell Membrane	Regulation of dopamine and 5-hydroxytryptamine levels in the brain, neuron activity, and behavior

## 4. Discussion

Our study applied a previous method isolating neuronally enriched microvesicle miRNAs using CD171 to serum samples of control and PD participants. Consistent with our hypotheses, we discovered a panel of smRNA expressions upregulated and downregulated in PD compared to control subjects. Four individual smRNAs in an LR model demonstrated a modest ability to discriminate between control and PD subjects (AUC ≥ 0.74). The 4 LR models showed that simple univariate models have some predictive ability and their performance serves as a baseline. The RF model of 29 smRNAs had high accuracy in distinguishing groups (AUC = 0.942). The identified smRNAs were involved in cell proliferation and DNA repair. The current findings suggest that neuronally-enriched microvesicle smRNAs may be identified in PD subjects and mark the disease. Further studies are warranted to validate these findings and explore pathological insights.

The predictive qualities of exosomal/microvesicle miRNAs for PD have been explored. miRNA was isolated from the serum of 30 PD patients and was compared to 30 healthy controls to test for the differential expression of ex-miRNAs in PD patients (Barbagallo et al., 2020). The expression levels of let-7d, miR-22* (asterisk indicates anti-sense miR), miR-23a, miR-24, miR-142-3p, and miR-222 were significantly increased in the serum of PD patients. In addition, receiver operating characteristic (ROC) curve analysis revealed that these six ex-miRNAs are ideal biomarkers to predict the PD phenotype. Another differential expression study compared the levels of 24 miRNAs from the serum of 109 PD patients and 40 healthy controls (Cao et al., 2017). The study showed that the levels of miR-24 (AUC, 0.908) and miR-195 (AUC, 0.697) were increased, and miR-19b (AUC, 0.753) was decreased in PD patients, indicating the possible use of miRNA as a novel strategy to ascertain PD status. Although the miRNAs from serum exosomes and microvesicles can be indicators of PD, the cellular origins of these vesicles and their respective miRNA cargo concerning PD are unknown. Several studies also have analyzed smRNA levels in body fluids (Margis et al., 2011; Mushtaq et al., 2016) (serum, plasma, and CSF) when comparing PD and healthy control patients, but the findings have been inconsistent (Li and Le, 2020). The discrepancies may involve differences in smRNA stability in samples (Blondal et al., 2013), analysis platforms (Roser et al., 2018), biofluids used (McDonald et al., 2011; Leggio et al., 2017), study populations (Leggio et al., 2017), and unknown cell type origins of smRNA.

To our knowledge, this is the first study analyzing the expression of neuronally-enriched smRNAs in serum samples from PD patients using next-generation sequencing, qRT-PCR validation, and evaluating the predictive value of the detected microvesicle smRNAs with LR and RF classifiers. Their involvement in regulating PD has been discussed (Filatova, 2012; Leggio et al., 2017). We observed that 29 neuronally-enriched exosomal smRNAs were expressed differentially between PD and control subjects; four of the 29 smRNAs align with previous findings. The smRNAs we identified have been implicated as circulating biomarkers in PD such as hsa-miR-26b-5p, hsa-miR-181a-5p, hsa-miR-221-3p, and miR-21-5p. In substantia nigra tissue, hsa-miR-26b-5p is upregulated in PD patients and neuronally derived exosomes and microvesicles from PD patients (Martinez and Peplow, 2017). Hsa-miR-26b-5p is involved in cell proliferation and apoptosis regulation for multiple myeloma, hepatocellular carcinoma, and subarachnoid hemorrhage phenotypes (Wang et al., 2016; Jia et al., 2018; Liu et al., 2021). Hsa-miR-181a-5p [midbrain neurons (Hegarty et al., 2018)] and hsa-miR-221-3p [blood plasma (Schulz et al., 2019; Chen et al., 2021)] are upregulated in PD patients. Previous studies suggest these smRNAs are dysregulated in PD and affect neuronal proliferation and apoptosis (Hamada et al., 2012; Hegarty et al., 2018; Jia et al., 2018). In agreement with a previous study in peripheral blood mononuclear cells from PD patients (Fu et al., 2017), miR-21-5p was upregulated in our study, which suggests an autoimmune response in PD. The exosomes and microvesicles captured in this study likely originated from neurons and may suggest that perturbations in the smRNA cargo reflect changes in neurons linked to the PD phenotype. Our finding indicates that the neuronally derived exosomes and microvesicles from blood serum carry known PD smRNAs previously detected directly from brain tissue and blood plasma.

Our smRNA differential expression analysis was consistent with some previous findings (Fu et al., 2017) but differed from other studies (Hamada et al., 2012; Fu et al., 2017). For example, previous studies reported that miR-221-3p and miR-181a-5p were downregulated in PD serum (Margis et al., 2011; Hamada et al., 2012; Ding et al., 2016). In contrast, we found that miR-221-3p and miR-181a- 5p were upregulated in PD cases versus controls. These discrepant results may have occurred due to differences in miRNA extraction methods (whole blood vs. serum). Previous studies have shown that smRNAs from whole blood exist outside exosomes and microvesicles (Ding et al., 2016) and smRNAs are more stable when contained within an exosome versus those freely circulating (Gallo et al., 2012; Zhang et al., 2015). Another factor may be differences in cellular sorting by smRNA type and quantity during the exosome loading (Zhang et al., 2015). Antiparksonian drugs have a documented effect on smRNA expression (Alieva et al., 2015; Goh et al., 2019) but our results lacked significance due to the small sample size and unbalanced groups. Future studies testing neuronally-enriched exosomal versus free-circulating smRNA profiles may provide further insight into the specific sample and procedure that best reflects PD pathogenesis.

In addition to differential expression, we further tested whether the smRNA profile may differentiate control and PD participants. Univariate LR of the smRNAs demonstrated a modest ability to discriminate between groups. The top 4 LR predictors were hsa-miR-6,073 [associated with small lung cancer (Kuang et al., 2020)], hsa_piR_016658 [upregulated in glioblastoma extracellular vesicles (Hallal et al., 2020)], hsa_piR_019825 [upregulated in glioma cell lines (Zimta et al., 2020)], and hsa-miR-21-5p [associated with oncogenic factor for glioblastoma (Jiang J. et al., 2020; Lu et al., 2020)]. These 4 smRNA predictors suggest a disruption in normal cell proliferation may indicate PD while sharing characteristics with phenotypes with altered cellular proliferation, such as glioblastoma. These univariate models assume independence from other smRNAs; however, smRNAs are prone to multicollinearity (Barbagallo et al., 2020). Although the smRNAs do not have high AUC values expected for diagnostic purposes (Hajian-Tilaki, 2013), they have better predictive abilities than random chance. Further molecular studies of these candidate biomarkers and their incorporation into more sophisticated multivariate prediction models may yield further insights.

The RF model using all 29 smRNAs indicated these factors had high accuracy (AUC = 0.942) in discriminating between control and PD subjects. This increase in model performance might be due to the biological complexity captured from multiple smRNA expression variables. Unlike the univariable LR models, RF models are tree-based and capture some non-linear dependencies among the 29 smRNA features (Menze et al., 2009). The top 10 smRNA predictors in the RF model were associated with breast cancer survival (Krishnan et al., 2016), colorectal cancer (Qu et al., 2019), germline-specific functions (Girard et al., 2006), and white matter lesions near lateral ventricles (Hamada et al., 2012). The top predictors in the RF models were associated with functions involving disruption of cell proliferation, consistent with the LR model results. This finding may further implicate perturbations in essential cell proliferation pathways associated with PD. The germline-specific smRNAs involve meiosis and stem cell maintenance (Cox et al., 2000), which may reflect changes in genomic stability. These results suggest a neuronal stress response involving cellular proliferation pathways may occur in PD.

The Gini importance calculations (Figure 3) ranked the predictors according to how much a feature decreases prediction error on average across RF models of different combinations of these 29 features. Although our RF model will require additional rigorous testing before being considered for diagnostic purposes, the smRNA predictors with the highest Gini importance values may be candidate markers. In the future, those smRNAs can be prioritized for detection in a clinical setting since they contribute more toward decreasing the classification error than smRNAs with the lowest Gini importance values. Additional studies are warranted to elucidate the role of these smRNAs in explaining how these markers contribute to PD pathophysiology.

Using LASSO followed by linear regression, however, we found no significant associations between smRNAs and clinical parameters (Table 3). LASSO aided in identifying smRNAs (from the set of 29 differentially expressed smRNAs) that were multicollinear with one another in the set by shrinking their coefficients to 0. The results suggest that smRNAs are prone to affect one another’s expression. A possible explanation for the lack of significant results is that PD patients have heterogenous clinical presentations (Foltynie et al., 2002; Lewis et al., 2005; Kehagia et al., 2010). The PD cohort in this study had a mean Hoehn and Yahr stage of 2.2 (SD = 1.12), indicating a mild disease stage (Goetz et al., 2004). The range was 1–5 (Table 2), with most PD participants having Hoehn and Yahr scores of 2. A prior study in a PD cohort with a mean Hoehn and Yahr score of 1.8 also reported no significant associations between miRNAs from cerebrospinal fluid and UPDRS and Hoehn and Yahr scores (Marques et al., 2017). Another study found that hsa-miR-4,639-5p was upregulated in early stage (Hoehn and Yahr scores 1–2.5) PD patients compared to controls, but there was a lack of significance for that same miRNA for those with Hoehn and Yahr scores ≥3 when compared to controls (Chen et al., 2017). These data suggest specific smRNAs may be better suited as a marker of disease rather than progression. Additional studies exploring broader motor and non-motor measures are needed to determine the potential clinical meaning of smRNA changes in PD.

The IPA was used to gain genetic context by identifying genes (Table 5) associated with the smRNAs expressed differentially (22 upregulated and 7 downregulated) in control and PD subjects. This included genes associated with annealing DNA strands and regulating pathways involved in Schwann cell myelination and apoptosis (e.g., *Tp53, DDIT4*) (Lu et al., 2017). The smRNAs also impact genes responsible for a neuronal calcium sensor (Burgoyne and Weiss, 2001) that modulates neuronal death (*VSNL1*) (Burgoyne and Weiss, 2001; Recabarren and Alarcón, 2017). The current findings for *Tp53*, *DDIT4*, and *VSNL1* are consistent with those reported previously, indicating they both participate in neuronal death in PD (Lu et al., 2017; Recabarren and Alarcón, 2017). *HTR1-A* has been proposed to be involved in dopamine regulation and the mechanistic pathway of antiparkinsonian drugs (Cacabelos, 2020). These data also suggest that smRNAs participation in PD manifestation may involve altered cell proliferation and apoptosis. There is accumulating evidence to link oncogenic and neurodegenerative processes to PD (Pan et al., 2008). We found candidate biomarkers of PD and contextualized their biological function, albeit with constraints.

Although our study identified smRNAs associated with PD, it still has limitations. Selectively isolating neuronally derived exosomes and microvesicles relies on immunoprecipitation using antibodies against CD171 that are not exclusively expressed on neurons. Although the exosome size was consistent with previous reports (Tomlinson et al., 2015), extracellular vesicles with a size of ≥200 nm also were isolated using the current methods. The isolated vesicles require validation in a future study via western blot with CD9, CD63, and CD171 antibodies (Kowal et al., 2017). The samples were collected and exosomes were extracted between 2012–2015; the freeze–thaw cycles and the long-term −80°C blood sample storage negatively affect exosome concentrations (Ge et al., 2014; Gelibter et al., 2022). An up-to-date exosome extraction from these blood samples would not represent the original exosomes extracted and sequenced. Freeze–thaw cycles and − 80°C storage also affect exosome morphology by promoting exosome fusion, increasing its size beyond 200 nm (Gelibter et al., 2022). Exosome fusion may also explain the presence of vesicles within the 200–400 nm range, according to Nanosight data. The exosome fusion will adversely affect the Western blot due to the size increase and the change in charge (Ge et al., 2014; Petersen et al., 2018). A suggested solution for future studies involves extracting exosomes from fresh blood samples before cold storage (Gelibter et al., 2022).

SmRNAs were the biomarkers of interest; however, exosome cargo such as fragments of RNA pseudogenes (e.g., RNA5SP382) was sequenced and those respective reads were aligned to pseudogenes (Raposo and Stoorvogel, 2013). SmRNAs are not the only cargo in the captured exosomes and microvesicles that can be sequenced; for example, messenger RNAs from serum exosomes have been used to characterize neurodegenerative diseases (Sproviero et al., 2022). Challenges in exosome isolation may affect the pathways identified by the IPA that was not specific to neurons. In addition, the association of smRNA findings with clinical metrics was explored. However, there was a lack of significance likely due to the heterogeneity of PD disease progression and the PD group representing an intermediate disease stage. Additional measures (e.g., non-antiparkinsonian drugs) should be investigated to obtain a more robust perspective of their impact. Medications can change miRNA expression profiles in PD subjects (Alieva et al., 2015), as shown by the common PD medication levodopa (Margis et al., 2011). Thus, further work is needed to confirm the predictive capabilities of the smRNAs identified, adjust for possible drug confounders, and optimize neuronal exosome isolation. Due to a lack of a validation set, our logistic regression and random forest models should be considered exploratory to find biological insights instead of a clinically applicable prediction model (Steyerberg, 2018).

This study employed a novel approach to isolate neuronally enriched exosomal smRNAs to identify those expressed differentially in control and PD subjects. Whereas a small number of smRNAs had a modest ability to discriminate controls from PD subjects, a combination of all 29 smRNAs expressed differently between groups had high accuracy. Although the smRNAs were not correlated with PD clinical metrics, they were associated with oncogenic and cellular proliferation processes. These data suggest smRNAs may have utility as PD biomarkers and the profiles we identified are distinct from those previously reported using serum or plasma circulating smRNA. The method of capturing exosomes and microvesicles and sequencing the smRNA cargo can be adapted to other complex neurological diseases to construct prediction models and detect new markers that also may be biologically informative.

## Data availability statement

The datasets presented in this study can be found in online repositories. The names of the repository/repositories and accession number(s) can be found at: https://www.ncbi.nlm.nih.gov/geo/, GSE167239.

## Ethics statement

The studies involving human participants were reviewed and approved by Penn State Hershey Institutional Review Board. The patients/participants provided their written informed consent to participate in this study.

## Author contributions

HM, ML, KV, XH, and YK: study concept. MA, SE, HM, ML, and VM: study execution. MA, HM, and VM: statistical analysis. MA, HM, and SE: manuscript preparation. ML, XH, MA, MH, and YK: review and critique. All authors contributed to the article and approved the submitted version.

## Funding

This work was supported by Penn State College of Medicine Junior Faculty develop program and Translational Brain Research Center pilot funding. This work also was supported by the USDA National Institute of Food and Agriculture and Hatch Appropriations under Project #PEN04275 and Accession #1018544, Seed funding from Penn State Institutes of Energy and the Environment (https://iee.psu.edu/), startup funds from the College of Agricultural Sciences, Pennsylvania State University (https://agsci.psu.edu/), and the Frances Keesler Graham Early Career Professorship from the Social Science Research Institute, Pennsylvania State University (https://ssri.psu.edu/) and also supported in part by the National Institute of Neurological Disorders and Stroke and Parkinson’s Disease Biomarker Program (NS060722 and NS082151 to XH) and the Penn State National Center for Advancing Translational Sciences, National Institute of Health, through the grant UL1 TR002014. MA was supported on the PSU/NIDDK-funded Integrative Analysis of Metabolic Phenotypes (IAMP) Predoctoral Training Program (T32DK120509).

## Conflict of interest

The authors declare that the research was conducted in the absence of any commercial or financial relationships that could be construed as a potential conflict of interest.

## Publisher’s note

All claims expressed in this article are solely those of the authors and do not necessarily represent those of their affiliated organizations, or those of the publisher, the editors and the reviewers. Any product that may be evaluated in this article, or claim that may be made by its manufacturer, is not guaranteed or endorsed by the publisher.

## Figures and Tables

**Table:** 

AUC	Area under the receiver operating characteristic curves
CPS	Counts per read
FDR	False discovery rate
HDRS	Hamilton Depression Rating Scale
IPA	Ingenuity Pathway Analysis
LASSO	Least absolute shrinkage and selection operator
LR	Logistic regression
MDS	Multidimensional scaling
MDS-UPDRS-III	Movement Disorders Society Unified PD Rating Scale motor subscale
miRNAs	Micro RNAs
MoCA	Montreal Cognitive Assessment
PD	Parkinson’s disease
piRNAs	Piwi-interacting RNAs
qRT-PCR	Quantitative real-time polymerase chain reaction
RF	Random forest
SmRNAs	Small RNAs
UPSIT	University of Pennsylvania Smell Identification Test

## Footnotes

^1^https://github.com/drpowell/degust
