# Supplementary figures and images for: Neuronally enriched microvesicle RNAs are differentially expressed in the serums of Parkinson’s patients
Source: Front Neurosci. 2023 Jul 6;17:1145923. doi: 10.3389/fnins.2023.1145923 (PMC10357515; doi:10.3389/fnins.2023.1145923)

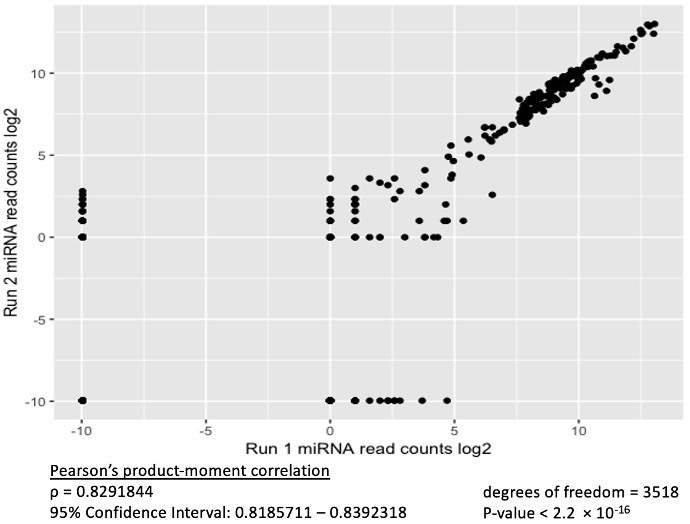

Supplement: Supplementary file 2 [file Image_1.TIFF]
